# Association between Dental Variables and Hashimoto's Disease: A Retrospective Cohort Study

**DOI:** 10.1055/s-0044-1800825

**Published:** 2025-03-12

**Authors:** Romana Peršić Bukmir, Ema Paljević, Jelena Vidas Hrstić, Elvis Božac, Katarzyna Mocny-Pachonska, Ivana Brekalo Pršo

**Affiliations:** 1 Department of Endodontics and Restorative Dentistry, Faculty of Dental Medicine, University of Rijeka, Rijeka, Croatia; 2 Division of Dentistry, Department of Conservative Dentistry with Endodontics, School of Medicine, Medical University of Silesia in Katowice, Bytom, Poland

**Keywords:** dental caries, Hashimoto disease, periapical periodontitis, periodontitis

## Abstract

**Objectives** The present study aimed to compare dental, endodontic, and periodontal status in patients with Hashimoto's disease and healthy patients, as well as to disclose the relation between dental variables and Hashimoto's disease.

**Materials and Methods** The research included 85 patients affected by Hashimoto's thyroiditis (analyzed group) and 85 healthy patients (control group). The two groups were matched according to age and gender. Data regarding patients' health status was acquired from their medical records. Data regarding dental, endodontic, and periodontal status were acquired from patients' dental records and digital panoramic radiographs.

**Statistical Analysis** Since a nonnormal distribution of data was detected, a median and interquartile range were used as a measure of central tendency and dispersion. Mann–Whitney *U* test and chi-squared test were used to test the differences between the groups. Multiple linear regression analysis and logistic regression analysis were used to test the association of the data.

**Results** Healthy participants had significantly higher median number of teeth with secondary caries (median 2; interquartile range 1–3) when compared with participants with Hashimoto's disease (median 1; interquartile range 0–2; *p* < 0.001). Periodontal disease was present in significantly more participants with Hashimoto's disease than healthy patients (68.2% vs. 45.9%; chi-square = 7.779; *p* = 0.005). The presence of Hashimoto's disease increased the risk of periodontal disease presence three times (odds ratio [OR] = 3.14; 95% confidence interval [CI] 1.38–7.15; *p* = 0.007). The presence of periodontal disease increased the risk of Hashimoto's disease presence by 2.5 times (OR 2.54; 95% CI: 1.36–4.73; *p* = 0.004).

**Conclusion** With the study limitations in mind, it may be concluded that a positive relationship between periodontal disease and Hashimoto's thyroiditis exists.

## Introduction

Plethora of epidemiological studies have reported associations between systemic health and oral inflammatory conditions such as periodontal disease (PD) and apical periodontitis (AP). 1 2 3 4 5 6 7 8 PD is a chronic inflammatory condition of tooth-supporting tissues characterized by alveolar bone loss and dysbiosis of oral microbiome. 9 It affects 10 to 15% of the population worldwide and studies have reported associations of PD with several chronic inflammatory-driven disorders such as cardiovascular, endocrine, neurodegenerative, and autoimmune. 1 2 9 10 11

AP is a chronic inflammatory disease of microbiological origin affecting periradicular tissues. According to the systematic review, 52% of world population has at least one tooth with AP. 12 13 Although many studies reported connection between factors inherent to endodontic treatment or postoperative coronal restoration and AP prevalence, the associations of periapical status and the general health of the patient were also indicated. 1 14 Impaired general health of patients and the diagnosis of systemic diseases like diabetes mellitus (DM), coronary heart disease, inflammatory bowel disease, rheumatoid arthritis, and psoriasis have been associated with a greater prevalence of AP. 1 3 4 5

Data regarding the connection between the thyroid dysfunction and oral inflammatory conditions are relatively scarce. Several studies indicated associations between PD and thyroid dysfunction, but without established causality. 6 7 8 Thyroid gland is a common target for a variety of disorders ranging from goiter to hyper- and hypothyroidism. 15 It is estimated that hypothyroidism affects around 5% of global population, with a further estimation of 5% being undiagnosed. 16 17 Environmental iodine deficiency is the most common cause of all thyroid disorders, including hypothyroidism. However, in areas of iodine sufficiency, Hashimoto's disease or chronic autoimmune thyroiditis is reported as the most common cause. Levothyroxine is the usual therapy prescribed for the management of hypothyroidism. 17

The thyroid gland is an important endocrine organ that secretes hormones and regulates growth and development of the body and its metabolic functions. Since it is anatomically and developmentally closely related to oral cavity, it has been hypothesized that infections from the oral cavity such as AP or PD may affect the thyroid gland. 6 Several studies reported significant association between dental status and thyroid disease emphasizing the role of dental practitioner in early screening of thyroid gland dysfunction as well as importance of controlling oral health conditions in patients with known thyroid disorders. 6 18

The aim of the present study was to compare dental, endodontic, and periodontal status in patients with Hashimoto's disease and healthy patients, and to disclose the relation between dental variables and Hashimoto's disease. Although the concept of the study is not novel, it brings complementary results to those of the previous research and augments the current knowledge on this important issue.

## Materials and Methods

The study protocol was approved by the ethics committee of the Clinical Hospital Center, Rijeka, Croatia (number of ethical approval 2170–29–02/1–23–2) and was performed in accordance with the Helsinki Declaration. The research was conducted according to the principles of cohort retrospective study. The medical, dental, and radiographic records of patients older than 18 years who had been referred to the Dental Clinic of the Clinical Hospital Center, Rijeka, Croatia between January 2023 and January 2024 were investigated.

The research included 85 patients affected by Hashimoto's thyroiditis (analyzed group) and 85 patients with no history of autoimmune disease or immunomodulatory therapy (control group) matched with analyzed group according to age and gender. Data regarding patients' health status was acquired from their medical records. All patients in the analyzed group were taking thyroxine replacement medications. Participants with DM type I or II, autoimmune disease other than Hashimoto's disease, patients taking medications known to alter immune response or bone metabolism, and/or history of smoking were excluded from both groups. The survey excluded participants that have not signed informed consent and agreed that their medical, dental, and radiographic records may be utilized for the purpose of research, as well as patients with incomplete medical, dental, or radiographic records.

For survey of dental, endodontic, and periodontal status, digital panoramic radiographs were analyzed on a 19-inch liquid crystal monitor (P1914S; Dell, Austin, Texas, United States; resolution: 1.280 × 1.024 32-bit color; graphic card: HD Graphic; Intel, Santa Clara, California, United States). Third molars were excluded from analysis. The following variables regarding dental status were recorded: number of remaining teeth, number of extracted teeth, number of decayed teeth (primary and secondary caries), number of restored teeth (filled or crowned), quality of coronal restoration, and number of teeth with inadequate restoration.

Caries and restoration quality assessment were based on clinical data acquired from patients' records and supplemented by findings on digital panoramic radiographs. Clinically, caries was recorded according to the World Health Organization criteria. Recurrent caries was defined as caries in a filled surface. Number of carious, missing, and filled teeth (Decayed, Missing, and Filled Teeth [DMFT]) was scored. 19 Quality of coronal restoration was recorded as 0 = adequate restoration or 1 = inadequate restoration, according to previously described clinical and radiological criteria. 20 21

The periapical status for each tooth was recorded utilizing the periapical index (PAI) scoring system by a calibrated observer. Kappa values for inter- and intraexaminer agreement were 0.70 and 0.75, respectively. Multirooted teeth were categorized according to the root with the highest score. PAI scores were dichotomized, and AP was recorded as absent (PAI scores 1 and 2) or present (PAI scores 3, 4, and 5). 22 Furthermore, periapical lesions were registered as related to a nonroot-filled tooth (primary AP) or to a root-filled tooth (secondary AP). Finally, the number of teeth with AP, the number of endodontically treated teeth, quality of root-canal filings, and the number of teeth with inadequate root canal fillings were recorded. Periapical disease ratio (number of teeth with AP divided by number of present teeth) was calculated. 23 Quality of root-canal filling was recorded as 0 = adequate or 1 = inadequate according to the criteria described by Song et al. 21

Periodontal status was recorded from panoramic radiographs. Proportion of the remaining bone height supporting each tooth was calculated according to the formula: *total bone height* (distance from marginal bone to the radiographic apex) / *total root length* (distance from the cemento/enamel junction to the radiographic apex × 100. 24 25 The *mean bone height* for the six Ramfjord teeth was determined to obtain one representative value for each patient. The Ramfjord teeth were considered representative for all 28 teeth. 24 Subsequently, the periodontal status was defined as *healthy* (≥ 80% remaining bone), *mild–moderate* (79–66%), or *severe PD* (< 66%). 24 25 For statistical analyses, the periodontal status was dichotomized into healthy and PD presence.

### Statistical Analysis

Statistical calculations were conducted using MedCalc statistical software (MedCalc Software Ltd., Ostend, Belgium). The level of statistical significance was set at *p* -value less than 0.05. Since testing the data for normality distribution by Lilliefors test demonstrated a nonnormal distribution, a median and interquartile range were used as a measure of central tendency and dispersion. To test the differences in the continuous variables between the two groups, Mann–Whitney *U* test was used. For determining the difference in frequencies between the groups, chi-squared test was utilized. Multiple linear regression analysis (backward model) was used to test the associations between independent variables and a periapical disease ratio, which was used as an outcome variable. Two models of multivariate logistic regression were used to determine predictors for periapical disease and Hashimoto's thyroiditis, respectively.

## Results

Eighty-five patients (78 women and 7 men) with Hashimoto's disease, median age 61 (interquartile range 54–70), were identified as eligible for the present survey. The control healthy group was similar according to age (median 61, interquartile range 54–71) and gender representation (78 women and 7 men; Table 1 ). The two groups had similar average number of present teeth, DMFT scores, average numbers of restored teeth, root-filled teeth, and teeth with AP ( Table 2 ). In the group with Hashimoto's disease, 85.9% of participants had at least one tooth with AP, while in the control group the prevalence of AP was 78.8% (chi-square = 0.077; *p* = 0.314). The prevalence of endodontic treatment in both groups was 85.9% (chi-square = 0.017; *p* = 0.826). The prevalence of root-filled teeth with AP was 72.9% in the Hashimoto's disease group and 67.1% in the control group (chi-square = 0.448; *p* = 0.503).

**Table 1:** The descriptive data of the sample

Variables		Healthy	Hashimoto	Test	*p*
Age (median, interquartile range)		61 (54–71)	61 (54–70)	Mann–Whitney *U* test	0.965
Gender	Male	7 (8.2%)	7 (8.2%)	Chi-squared test	0.780 Chi-square = 0.078
	Female	78 (91.8%)	78 (91.8%)		

**Table 2:** The differences in dental variables between patients with Hashimoto's disease and healthy patients

Variables		Healthy	Hashimoto	Test	Statistics
*N* present teeth (mean, interquartile range)		22 (17–26)	20 (15–25)	Mann–Whitney *U* test	*p* = 0.139
DMFT (mean, interquartile range)		20 (16–22)	21 (15–25)	Mann–Whitney *U* test	*p* = 0.328
*N* primary caries (mean, interquartile range)		0 (0–1)	0 (0–2)	Mann–Whitney *U* test	*p* = 0.525
*N* secondary caries (mean, interquartile range)		2 (1–3)	1 (0–2)	Mann–Whitney *U* test	*p* < 0.001 a
*N* restored teeth (mean, interquartile range)		10 (7–14)	10 (6–12)	Mann–Whitney *U* test	*p* = 0.297
*N* teeth with inadequate restorations (mean, interquartile range)		3 (1–5)	2 (1–5)	Mann–Whitney *U* test	*p* = 0.075
*N* teeth with apical periodontitis (mean, interquartile range)		2 (1–4)	2 (2–3)	Mann–Whitney *U* test	*p* = 0.925
*N* root-filled teeth (mean, interquartile range)		3 (1–5)	2 (1–3)	Mann–Whitney *U* test	*p* = 0.064
*N* teeth with inadequate root canal filling (mean, interquartile range)		2 (0–4)	1 (0–2)	Mann–Whitney *U* test	*p* = 0.074
*N* root-filled teeth with apical periodontitis (mean, interquartile range)		1 (0–3)	2 (0–2)	Mann–Whitney *U* test	*p* = 0.736
Prevalence of apical periodontitis		67 (78.8%)	73 (85.9%)	Chi-squared test	*p* = 0.314 Chi-square = 0.077
Prevalence of root-canal treatment		73 (85.9%)	73 (85.9%)	Chi-squared test	*p* = 0.826 Chi-square = 0.017
Prevalence of root-filled teeth with AP		57 (67.1%)	62 (72.9%)	Chi-squared test	*p* = 0.503 Chi-square = 0.448
Periapical disease ratio (number of teeth with AP divided by number of present teeth)		0.130 (0.038–0.232)	0.133 (0.041–0.222)	Mann–Whitney *U* test	*p* = 0.815
Periodontal status	Healthy	46 (54.1%)	27 (31.8%)	Chi-squared test	*p* = 0.005 a Chi-square = 7.779
	Periodontal disease	39 (45.9%)	58 (68.2%)		

Abbreviations: AP, apical periodontitis; DMFT, Decayed, Missing, and Filled Teeth.

a Statistically significant.

It was observed that healthy participants had significantly higher median number of teeth with secondary caries (median 2; interquartile range 1–3) when compared with participants with Hashimoto's disease (median 1; interquartile range 0–2; *p* < 0.001). Also, the difference in proportion of PD was observed between the two groups (chi-square = 7.779; *p* = 0.005). PD was present in 68.2% of participants with Hashimoto's disease. Conversely, only 45.9% of healthy participants had radiological signs of PD ( Table 2 ).

Multiple linear regression analysis (backward model) was utilized to identify predictors of AP. Periapical disease ratio was used as an outcome variable. Different predictor variables were entered into the multivariate model. The variables presenting the best fit are reported in Table 3 . Variables not included in the final model were *N* restored teeth, *N* inadequate restorations, gender (0 = male; 1 = female), Hashimoto's disease (0 = no; 1 = yes), and *N* secondary caries. Age, presence of PD, number of teeth having inadequate root canal filling, and number of teeth affected with primary caries are variables significantly associated with AP. They explained 40.4% of the observed variability in periapical disease ratio ( *R* ^2^ = 0.404; *p* < 0.001). The number of primary carious lesions accounted for a major part of variability (unique contribution 11.5%), followed by the number of inadequate root canal fillings (7.9%) and age (7%). PD accounted for a lower portion of variability (2.8%). The presence of PD increased periapical disease ratio by 0.048, while increase in number of primary caries and inadequate root canal fillings for one increased periapical disease ratio by 0.028 and 0.025, respectively.

**Table 3:** Association of independent variables and periapical disease ratio

Variable	*B*	SE	*p*	Sr
(Constant)	–0.144			
Age	0.003	0.001	< 0.001	0.265
Periodontal disease 0 = no 1 = yes	0.048	0.022	0.031	0.167
*N* root-filled teeth	0.001	0.006	0.091	0.131
*N* teeth with inadequate root canal filling	0.025	0.007	< 0.001	0.281
*N* primary caries	0.028	0.006	< 0.001	0.339

Abbreviations: *B* , unstandardized regression coefficient; SE, standard error of *B* coefficient; Sr, semipartial coefficient of correlation indicates the unique contribution to periapical disease ratio.

Note: Explained variance ( *R* ^2^ = 0.404.

Table 4 demonstrates the results of multivariate logistic regression (backward model). Variables not included in the model were gender (0 = male; 1 = female), *N* teeth with inadequate restorations, *N* secondary caries, and *N* of teeth with AP. Variables age (odds ratio [OR] 1.05; 95% confidence interval [CI] 1.01–1.10; *p* = 0.012) and number of restored teeth (OR 1.12; 95% CI 1.01–1.24; *p* = 0.027) demonstrated slightly increased but significant risk for PD presence. The presence of Hashimoto's disease demonstrated the highest association, increasing the risk of PD presence three times (OR = 3.14; 95% CI 1.38–7.15; *p* = 0.007). It was found that an increased number of present teeth reduces the risk for PD (OR 0.82; 95% CI 0.75–0.90; *p* < 0.001).

**Table 4:** Association of independent variables and periodontal disease

Variable	*β*	Standard error	*p* -Value	Odds ratio (95% confidence interval)
Age	0.05	0.02	0.012 a	1.05 (1.01–1.10)
Hashimoto 0 = no 1 = yes	1.14	0.42	0.007 a	3.14 (1.38–7.15)
*N* primary caries	0.33	0.17	0.053	1.39 (1.00–1.94)
*N* restored teeth	0.11	0.05	0.027 a	1.12 (1.01–1.24)
*N* present teeth	–0.20	0.05	< 0.001 a	0.82 (0.75–0.90)

a Statistically significant.

When age, gender, number of present teeth, number of teeth with AP, active carious lesions, and the presence of PD as predictor variables were tested against Hashimoto's disease (0 = no; 1 = yes) as an outcome variable in multivariate logistic regression, only PD presence remained significantly associated with Hashimoto's disease (OR 2.54; 95% CI: 1.56–4.73; *p* = 0.004). The presence of PD increased the risk of Hashimoto's disease presence by 2.5 times.

## Discussion

The present survey aimed to compare dental, endodontic, and periodontal status in patients with Hashimoto's disease and healthy patients. No difference was observed in dental status between the two groups, except for average number of teeth with secondary caries. Interestingly, healthy participants had significantly higher median number of teeth with secondary caries than participants with Hashimoto's disease. Although the scope of the present study did not include investigations of oral health behavior variables, previously conducted study reported association of thyroid diseases with good oral health behaviors, such as frequency of teeth brushing or use of oral hygiene products. 6 Therefore, it can be speculated that patients with Hashimoto's disease have better oral hygiene and less recurrent caries than the control group.

**Fig. 1:**
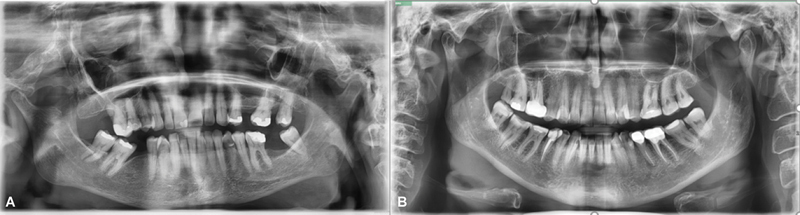
Panoramic radiographs of two 61-year-old female patients: ( **A** ) patient with Hashimoto's thyroiditis demonstrating moderate periodontal disease, and ( **B** ) healthy patient without radiological signs of periodontal disease.

**Fig. 2:**
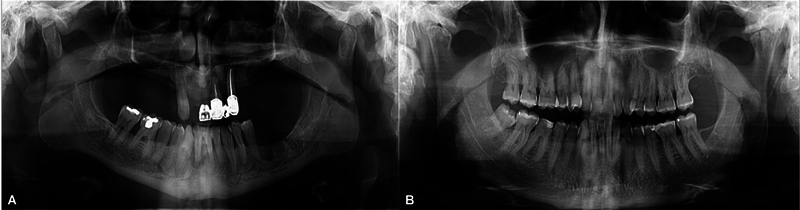
Panoramic radiographs of two 61-year-old male patients: ( **A** ) patient with Hashimoto's thyroiditis demonstrating severe periodontal disease, and ( **B** ) healthy patient without radiological signs of periodontal disease.

Figs. 1 and 2 illustrate the differences in radiologically determined periodontal status in subjects with Hashimoto's thyroiditis and their healthy counterparts. Our results demonstrated increased prevalence of PD in patients with Hashimoto's disease when compared with the control group (68% vs. 45%, respectively). This is in accordance with findings of a recent cross-sectional study that reported connection of high Community Periodontal Index with the abnormalities of thyroid function tests. 6 Several previous studies described relationship between hypothyroidism and moderate to advanced periodontitis and periimplantitis. 26 27 28

A decreased bone turnover rate has been observed in hypothyroid patients, possibly caused by reduced osteoclastic and osteoblastic activity. 29 Consequently, it may be hypothesized that AP healing in patients with hypothyroidism can be delayed. The present results demonstrated that the prevalence of root-filled teeth with AP was higher in the Hashimoto's disease group than in the control group (73% vs. 67%, respectively); however, the difference was not statistically significant. All participants with Hashimoto's disease in the present study had substitutional therapy. As levothyroxine therapy promotes normal bone mineral density in adults, 30 it may be speculated that this effect reduced the influence of the disease on the periapical status.

Predictors for periapical and PD were investigated through multivariate models as they best reflect biological conditions. In this survey the periapical disease ratio was used to express individual experience of periapical disease, rather than binary variable such as presence or absence of AP in participant that may underestimate the burden of periapical disease. 23 Multiple linear regression analysis did not reveal associations of periapical disease and Hashimoto's thyroiditis. Variables age, presence of PD, number of teeth having inadequate root canal filling, and number of teeth affected with primary caries were found to be significant predictors of periapical disease explaining 40.4% of the observed variability in the periapical disease ratio. These results are in line with the reports from previous studies that have identified similar variables as risk indicators for AP. 23 31

Although age and number of restored teeth were variables predictive for PD, the multivariate model demonstrated that Hashimoto's disease is the variable that brings the highest risk increasing the odds for PD presence by three times. This association could be explained by the hormonal imbalance and its effect on bone turnover. Generally, the homeostasis of the bone tissue involves a balance between processes of bone formation and resorption, which are conducted by osteoblasts and osteoclasts. 32 Periodontal bacterial infection and consequential immune responses can suppress differentiation and induce apoptosis of osteoblasts, and therefore contribute to osteoblastic cell loss. On the other hand, the inflammatory cascade, involving mechanisms of cellular and humoral immunity, contributes to osteoclastogenesis and alveolar bone loss. 32 Thyroid hormones have impact on remodeling of the bone by direct stimulation of osteoblasts and osteoclasts. 33 Hypothyroidism affects bone healing by reduction in recruitment, maturation, and activity of bone cells that can lead to reduction of bone formation and resorption. 33 In hypothyroidism, the resorption depth is reduced, and the completed wall thickness of the osteon, the bone structural unit, is increased. 33

Patients with Hashimoto's disease exhibit elevated levels of thyrotropin hormone (THS). It has been shown that THS regulates bone metabolism independently of thyroid hormones, including the function of osteoblasts and osteoclasts. 34 A recent *in vitro* study observed that THS could inhibit the osteogenic differentiation of periodontal ligament stem cells (PDLSCs) and thus negatively affect periodontitis prevalence and outcome. 34 The conclusion of the same study was that reducing serum TSH levels may improve the osteogenic differentiation of PDLSCs. Therefore, reduction in high TSH levels by administering low doses of thyroxine may be beneficial for patients with subclinical hypothyroidism who require an orthodontic, implant, or periodontal treatment. 34 It may be speculated that substitutional therapy by synthetic hormones such as levothyroxine may have similar effect. Furthermore, thyroid dysfunction is a condition that can facilitate the production of proinflammatory cytokines like interleukin-6 and tumor necrosis factor-α. 35 These cytokines can promote the production of proinflammatory agents like metalloproteinases resulting in osteoclast activation and destruction of connective tissue. 36 The associations between PD and thyroid dysfunction were investigated in previous surveys, but the causality was not established. 6 7 8

Hypothyroidism is a common condition that often coexists with other systemic disorders, such as obesity. This combination can have a synergistic effect on periodontal health through the facilitation of chronic inflammation and altered immune response. A recent study disclosed obesity as a risk factor for hypothyroidism, 37 with studies indicating that higher body weight correlates with increased alveolar bone loss. 38 The role of adipose tissue is significant in this relationship, as it modifies the secretion and activity of various cytokines and adipokines. Notably, leptin and adiponectin can inhibit osteoblast production while promoting osteoclast activity, leading to bone resorption. 39 Moreover, obesity is associated with a state of chronic systemic inflammation, which affects the periodontium. Elevated levels of proinflammatory cytokines and matrix metalloproteinases contribute to osteoclastogenesis, exacerbating bone loss and periodontal inflammation. 40

A recent study reported that PD should not be attributed to variations in thyroid function, nonetheless, it may have causal effect on hypothyroidism risk. 41 The link between PD and thyroid dysfunction is also observed in the findings of this study where the presence of PD increased the risk for Hashimoto's disease by 2.5 times. This finding stresses the importance of oral health maintenance since a study by Bhankhar et al reported a significant reduction in mean values of TSH following nonsurgical periodontal therapy. 42

Previous research demonstrated high prevalence of thyroid disease in women, 6 and the present survey confirmed this finding since 88% of the participants in the Hashimoto's disease group were women. Limited sample size and retrospective nature of the study design may present a limitation of this research, but the exclusion of important confounding factors presents an advantage. Patients with DM, autoimmune diseases other than Hashimoto's thyroiditis, patients taking medications that alter the immune response or bone metabolism, and smokers were excluded from the sample since all these conditions may act as confounding factors in the present investigation. It has been reported that smoking and DM are closely related to thyroid disfunction. 43 44 Furthermore, these conditions have also been recognized as risk factors for oral diseases such as AP and PD. 1 Nonetheless, to investigate a possible causal-effect relationship between oral variables and Hashimoto's disease a prospective study design should be applied. Besides exclusion of confounding factors, the influence of variables such as the duration of the disease and duration of substitutional therapy should be analyzed.

It is important to investigate the association between the status of the oral health and immune-related diseases. 6 An association between oral lichen planus and Hashimoto thyroiditis has been demonstrated emphasizing a significant relationship between immune response in oral cavity and thyroid disease. 45 Considering the associations between the oral health status and thyroid gland status reported by previous as well as current study, controlling of oral health conditions may be significant in early detection of patients at high risk for diseases related to thyroid gland. 6

## Conclusion

With the study limitations in mind, it may be concluded that a positive relationship between PD and Hashimoto's thyroiditis exists; however, further investigations are necessary to elucidate the underlying mechanisms and causality of this finding. Nonetheless, these findings have clinical importance, since early diagnosis and treatment of Hashimoto's disease and periodontitis can mutually improve outcomes for both conditions.
